# Renoprotective effects of green tea

**Published:** 2013-07-01

**Authors:** Shabnam Hajian

**Affiliations:** Medical Plants Research Center, Shahrekord University of Medical Sciences, Sharekord, Iran

**Keywords:** Oxidative stress, Green tea, Renoprotection

**Implication for health policy/practice/research/medical education:**

Green tea (Camellia sinensis) is a popular herbal remedy worldwide. Polyphenols in green tea have attracted much attention as potential compounds for the maintenance of human health due to their varied biological activities and low toxicities.

Free radicals lead to chronic diseases such as cancer, diabetes, atherosclerosis, nephrotoxicity, hepatotoxicity (1,2). Green tea (*Camellia sinensis*) is a popular herbal remedy worldwide. Polyphenols in green tea have attracted much attention as potential compounds for the maintenance of human health due to their varied biological activities and low toxicities. Recently the antioxidant properties of green tea on injury protection caused by oxidative stress have been on focus (3,4). Green tea, has antioxidant and anti-inflammatory properties (5). The antioxidant and anti-inflammatory properties of this herb is due to its polyphenolic compounds and flavonols such as catechins, gallic acid and phenolic acids. Catechin has strong antioxidative activity, which has been demonstrated by its ability to scavenge free radicals, inhibits free radical generation and chelates transition metal ions that catalyze free radical reactions (6-8). Antioxidant property of green tea is associated with formation of intra-molecular hydrogen bonds, depolarization of electrons and rearrangement of the molecular structure (2). Over the last years, abundant epidemiological studies have shown several physiological properties of green tea which may be relevant to the treatment and promotion of health in some chronic diseases (9). Meki *et al*, conducted a study to investigate the biochemical and histopathological effects of lead toxicity on liver, kidney and brain of rats. Moreover, the antioxidative activity of green tea extract against oxidative stress induced by lead toxicity was evaluated. The chelating property of green tea extract to reduce lead in rat tissues was detected (6). Salem and colleagues demonstrated that green tea extract was able to ameliorate gentamicin-induced nephrotoxicity and oxidative damage by improving antioxidant defense and tissue injury. However, further clinical studies are necessary to understand the antioxidant effects of green tea extract on kidney diseases. Indeed, green tea is an inexpensive, nontoxic remedy that can consume to prevent a risk for gentamicin -induced nephrotoxicity (10).

It has been reported that cyclosporine A caused loss of brush border and dilatation of proximal tubules, vacuolization, tubular atrophy, calcification, apoptosis, renal fibrosis and finally increased serum creatinine. Green tea polyphenols notably decreased cyclosporine A-induced kidney damage and improved kidney function (11). The result of our recent experimental study to test the efficacy of green tea extract against contrast media induced renal injury in Wistar rats indicated that green tea had protective effect against contrast media nephrotoxicity (12). According to our findings and previous studies on protecting effects of green tea against tubular injury, we can conclude that antioxidants are responsible for having renoprotective efficiency of this herb (12). In fact, our data is an evidence that green tea has antioxidant property to protect tubular renal cells. In this regard, to understand other properties of the green tea more experimental or clinical trials studies are suggested.

## Conclusion

According to our results and previous studies green tea has a renoprotective capability.

## Conflict of interests

The author declared no competing interests.

## Ethical considerations

Ethical issues (including plagiarism, data fabrication, double publication) have been completely observed by the author.

## Funding/Support

None.

## Author’s contribution

SH was the single author of the paper.
